# Modeling biological and genetic diversity in upper tract urothelial carcinoma with patient derived xenografts

**DOI:** 10.1038/s41467-020-15885-7

**Published:** 2020-04-24

**Authors:** Kwanghee Kim, Wenhuo Hu, François Audenet, Nima Almassi, Aphrothiti J. Hanrahan, Katie Murray, Aditya Bagrodia, Nathan Wong, Timothy N. Clinton, Shawn Dason, Vishnu Mohan, Sylvia Jebiwott, Karan Nagar, Jianjiong Gao, Alex Penson, Chris Hughes, Benjamin Gordon, Ziyu Chen, Yiyu Dong, Philip A. Watson, Ricardo Alvim, Arijh Elzein, Sizhi P. Gao, Emiliano Cocco, Alessandro D. Santin, Irina Ostrovnaya, James J. Hsieh, Irit Sagi, Eugene J. Pietzak, A. Ari Hakimi, Jonathan E. Rosenberg, Gopa Iyer, Herbert A. Vargas, Maurizio Scaltriti, Hikmat Al-Ahmadie, David B. Solit, Jonathan A. Coleman

**Affiliations:** 1 Department of Surgery, Memorial Sloan Kettering Cancer Center, New York, NY 10065 USA; 2 Human Oncology and Pathogenesis Program, Memorial Sloan Kettering Cancer Center, New York, NY 10065 USA; 3 Urology Service, Department of Surgery, Memorial Sloan Kettering Cancer Center, New York, NY 10065 USA; 4 Department of Biological Regulation, Weizmann Institute of Science, Rehovot, 7610001 Israel; 5 Marie-Josée and Henry R. Kravis Center for Molecular Oncology, Memorial Sloan Kettering Cancer Center, New York, NY 10065 USA; 6 Gynecology & Reproductive Sciences, Department of Obstetrics, Yale University School of Medicine, New Haven, CT 06510 USA; 7 Department of Epidemiology-Biostatistics, Memorial Sloan Kettering Cancer Center, New York, NY 10017 USA; 8 Molecular Oncology, Department of Medicine, Siteman Cancer Center, Washington University, St. Louis, MO 63110 USA; 9 Genitourinary Oncology Service, Department of Medicine, Memorial Sloan Kettering Cancer Center, New York, NY 10065 USA; 10 Body Imaging Service, Department of Radiology, Memorial Sloan Kettering Cancer Center, New York, NY 10065 USA; 11 Department of Pathology, Memorial Sloan Kettering Cancer Center, New York, NY 10065 USA

**Keywords:** Cancer, Cancer, Oncology, Oncology

## Abstract

Treatment paradigms for patients with upper tract urothelial carcinoma (UTUC) are typically extrapolated from studies of bladder cancer despite their distinct clinical and molecular characteristics. The advancement of UTUC research is hampered by the lack of disease-specific models. Here, we report the establishment of patient derived xenograft (PDX) and cell line models that reflect the genomic and biological heterogeneity of the human disease. Models demonstrate high genomic concordance with the corresponding patient tumors, with invasive tumors more likely to successfully engraft. Treatment of PDX models with chemotherapy recapitulates responses observed in patients. Analysis of a HER2 S310F-mutant PDX suggests that an antibody drug conjugate targeting HER2 would have superior efficacy versus selective HER2 kinase inhibitors. In sum, the biological and phenotypic concordance between patient and PDXs suggest that these models could facilitate studies of intrinsic and acquired resistance and the development of personalized medicine strategies for UTUC patients.

## Abstract (web summary)

The advancement of upper tract urothelial carcinoma (UTUC) research is hampered by the lack of disease-specific models. Here, the authors report patient derived xenograft and cell line models of UTUC, and show that these models retain the genomic and biological heterogeneity of human disease.

## Introduction

Upper tract urothelial carcinoma (UTUC) is a rare malignancy of the renal pelvis or ureter. Although it arises from the same urothelium and shares similarities to urothelial carcinoma of the bladder (UCB), UTUC has distinct molecular characteristics that are of clinical significance. Unlike UCB, UTUC is a Lynch Syndrome-associated malignancy, with the most recent study demonstrating 8.7% of UTUC patients having a pathogenic germline mutation in a Lynch Syndrome-associated gene^1^. Given the relative rarity of this cancer type, treatment guidelines such as the recommendation for the use of neoadjuvant chemotherapy are often extrapolated from UCB studies.

Recent genomic analyses of UTUC^2–5^ have identified a high incidence of potentially actionable genomic alterations including recurrent activating mutations in receptor tyrosine kinases (*FGFR3, ERBB2*), *HRAS*, *PIK3CA* and *TSC1*. These molecular profiling studies have also, however, identified significant genetic diversity among UTUC patients and a large number of genomic variants of unknown significance^6,7^. Patient-derived UTUC models would facilitate the biological characterization of these variants of unknown significance and could also be used as preclinical models to test novel precision therapy approaches. Although PDX models have been developed from patients with UCB^8–10^, the development of UTUC PDX models has yet to be reported. Similarly, although there are several dozen bladder cancer cell lines in widespread use^11,12^, few are of upper tract origin. The lack of UTUC-specific cell line and animal models is thus a major hurdle to the development of more effective treatment strategies for this disease.

Here, we report on the successful development of UTUC models that reflect the genetic and biological heterogeneity of the human disease. In a subset of PDX models, chemotherapy sensitivity mirrors the drug sensitivity observed in the patients from which they were derived. Although HER2 alterations are common in patients with urothelial cancer, HER2-targeted therapies have had only modest clinical impact in this disease. We thus used a HER2-mutant PDX/PDC model to explore the biological basis for the limited clinical activity observed to date with HER2-directed therapies in patients with urothelial carcinoma.

## Results

### Integrated genomic and transcriptomic analysis of UTUC

With the goal of facilitating the development of personalized therapeutic approaches for patients with UTUC, we began in 2014 offering prospective clinical tumor sequencing using the MSK-IMPACT assay to patients with UTUC^13^. MSK-IMPACT is a targeted capture-based next-generation sequencing (NGS) assay that can detect mutations, copy number alterations and select gene fusions in up to 468 cancer-associated genes^6^. To define the frequency of potentially actionable genomic alterations in UTUC, we analyzed 37 UTUC patients from this prospectively generated cohort and 82 UTUC patients previously analyzed retrospectively^2^. In total, tumor samples from 119 UTUC patients (118 nephroureterectomy and 1 resection of metastasis) were analyzed (Supplementary Fig. 1). Consistent with prior studies in UTUC^2,3^, we observed frequent mutations in potentially actionable genes including *FGFR3* (47%), *ERBB2* (9%), *HRAS* (12%), *PIK3CA* (16%) and *TSC1* (14%).

**Fig. 1:**
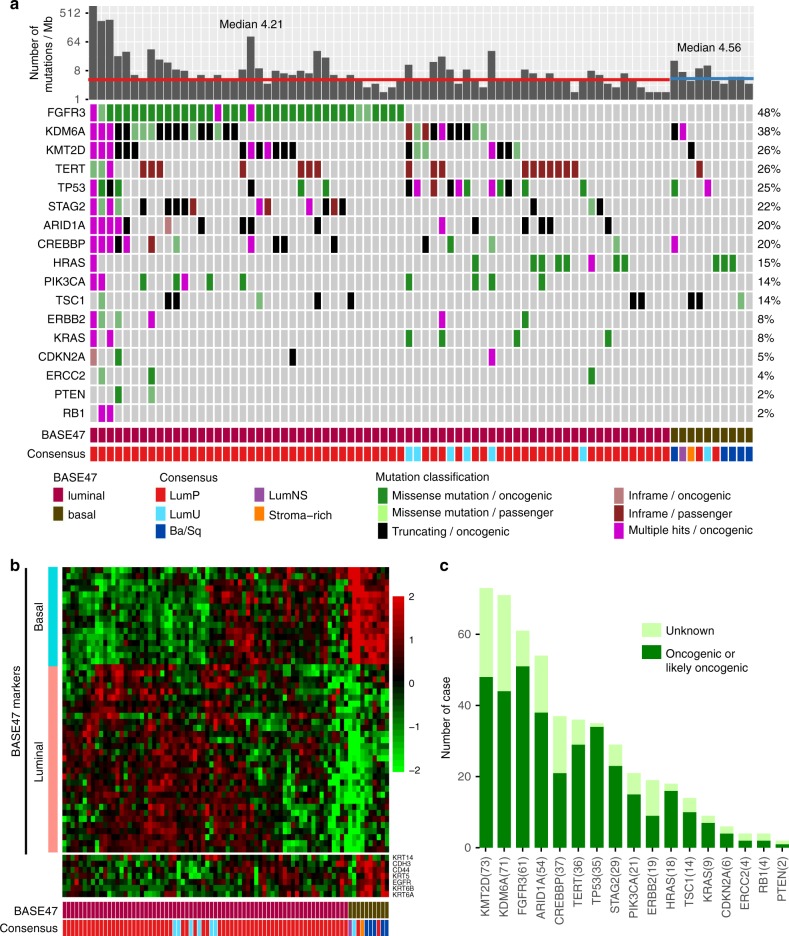
Integrated genomic and transcriptomic analysis of UTUC cancers. **a** Somatic genomic landscape of 80 UTUC tumors analyzed by MSK-IMPACT sequencing stratified by RNA sequencing analysis into luminal-like and basal-like subtypes defined using the BASE47 classifier (*K* = 2)^15^, and 5 subtypes based on a consensus molecular classification of muscle-invasive bladder cancer^16^. Tumor mutation burden per megabase (Mb) is indicated in log2 scale for each sample. **b** Clustering analysis of RNA-seq data from 80 UTUC tumor samples based on the BASE47 classifier (basal marks shown as a turquoise vertical bar; luminal marks shown as a salmon vertical bar). Analysis of genes expressed in cancer cells of a basal-like phenotype (*KRT14, CDH3, CD44, KRT5, EGFR, KRT6A/B*) is also shown (color key on the top of **b**). **c** The number of tumors with oncogenic/likely oncogenic and variants of unknown significance in 17 genes frequently mutated in UTUC. Source data for **a** and **b** are provided as a Source Data file.

Gene expression profiling analyses of muscle-invasive bladder cancers have identified basal and luminal subtypes with the basal sub-type associated with a more aggressive disease course^14,15^. To determine whether UTUC tumors can be similarly stratified, we performed whole-transcriptome RNA sequencing (RNA-seq) (Fig. 1a, b) on 80 of the 119 UTUC tumors for which MSK-IMPACT data was available. Patient demographic and clinical information for the RNA-seq cohort are reported in Supplementary Table 1. Clustering analysis based on the BASE47 gene classifiers^15^ found that 70 tumors (87.5%) had a luminal phenotype and 10 (12.5%) a basal phenotype (Fig. 1b). In addition, application of a consensus classifier developed by the Bladder Cancer Molecular Taxonomy Group^16^ revealed that the majority of UTUC in the cohort were luminal–papillary (LumP, 66 tumors, 82.5%) sub-type including all 14 of the low-grade tumors. The remainder were classified as luminal unstable (LumU, 7 tumors, 8.75%), luminal non-specific (LumNS, 1 tumor, 1.25%), Stroma-rich (1 tumor, 1.25%) and basal/squamous type (Ba/Sq, 5 tumors, 6.25%). The latter had high expression of tumor basal markers including *CDH3* (Cadherin-3), *CD44* (CD44 antigen), *KRT5* (Keratin, type II cytoskeletal 5), *EGFR* and *KRT6* (Keratin, type II cytoskeletal 6) present in 4 of 5 of the Ba/Sq-type tumors. There was no significant sub-type difference between high- and low-grade tumors (*P* = 0.5389, chi-square test).

Next, we explored differences in the frequency of common driver mutations in UTUC tumors with basal or luminal phenotypes as defined by the BASE47 classifier^15^. The median tumor mutation burden was 4.21 (1.7–853.2) for the luminal and 4.56 (3.1–16.4) for the basal phenotype tumors (Fig. 1a, *P* = 0.89). Somatic *FGFR3* mutations, which have previously been associated with a favorable prognostic outcome in UTUC^17^, were only present in luminal subtype. Conversely, there were no significant differences among the two subtypes in the percentage of patients with mutations in *TP53* or other driver genes commonly present in UTUC. Finally, using a curated knowledge base of the known biological effects of individual mutant alleles^18^, we observed that 39.3% of all somatic mutations identified were variants of unknown functional significance (Fig. 1c).

### Establishment and characterization of UTUC PDX and PDC

With the goal of exploring the biological and clinical significance of individual mutational events identified in the UTUC cohort, we leveraged our prospective clinical sequencing initiative to develop models of UTUC that reflect the genomic and biological diversity of the human disease. Surgical specimens primarily obtained following radical nephroureterectomy (RNU) were grafted into immunocompromised NOD *scid* gamma (NSG) mice to generate patient-derived xenograft (PDX) models with a subset also cultured in vitro to develop patient-derived cell line (PDC) models. In total, we successfully established 17 PDX models from 34 UTUC tumors (50% take rate). The tumor fragments at early passages of 16 among 17 PDX models were successfully preserved as frozen stocks for future implantation (Supplementary Table 2) to avoid late passage failure in tumorigenicity. Six PDC models among 24 tumors (6/24: take rate 25%) also survived beyond 10 passages (Supplementary Fig. 2). Although not statistically significantly different, we did observe a trend towards PDX growth in tumors that were muscle-invasive (≥pT2 tumor stage, *P* = 0.166) (Table 1). All three tumors collected from distant (UCC3 [abdominal wall] and UCC11 [liver]) or lymph node (UCC32) metastatic sites successfully engrafted. The only tumor collected at endoscopic biopsy (UCC27) did not engraft.

**Table 1:** Clinico-pathological characteristics of the samples used for UTUC PDX.

Variable	PDX growth (*N* = 17)	No PDX growth (*N* = 17)	*P*-value
Median age at surgery, years (IQR)	69.6 (65.7–75.7)	65.6 (60.0–75.3)	0.390
Gender			1.000
Male	11 (64.7)	11 (64.7)	
Female	6 (35.3)	6 (35.3)	
Lynch syndrome/MSI-H			0.166
Yes	4 (23.5)	1 (5.9)	
No	12 (70.6)	16 (94.1)	
Not tested	1 (5.9)	0 (0)	
Smoking status			0.039
Current/former	10 (58.8)	16 (94.1)	
Never	7 (41.2)	1 (5.9)	
Prior history of urothelial cancer			1.000
Yes	5 (29.4)	6 (35.3)	
No	12 (70.6)	11 (64.7)	
Location of primary tumor			1.000
Renal pelvis	15 (88.2)	14 (82.4)	
Ureter	2 (11.8)	3 (17.6)	
Specimen type			0.6^a^
Primary	14 (82.4)	16 (94.1)	
Lymph node	1 (5.9)	0 (0)	
Distant metastasis	2 (11.8)	0 (0)	
Endoscopic biopsy	0 (0)	1 (5.9)	
Grade			0.480
Low	2 (11.8)	0 (0)	
High	15 (88.2)	17 (100)	
pT stage			0.166
<pT2 (non-invasive)	5 (29.4)	10 (58.8)	
≥pT2 (invasive)	12 (70.6)	7 (41.2)	
pN stage			0.460
pN0	10 (58.8)	13 (70.5)	
pN+	5 (29.4)	2 (11.8)	
pN*x*	2 (11.8)	2 (11.8)	
Prior chemotherapy			0.688
Yes	3 (17.6)	5 (29.4)	
No	14 (82.4)	12 (70.6)	

Two-sided *P*-values were obtained using Wilcoxon-rank sum for continuous and Fisher’s exact test for categorical variables.

^a^Specimen type was dichotomized as primary vs all others for this comparison.

**Fig. 2:**
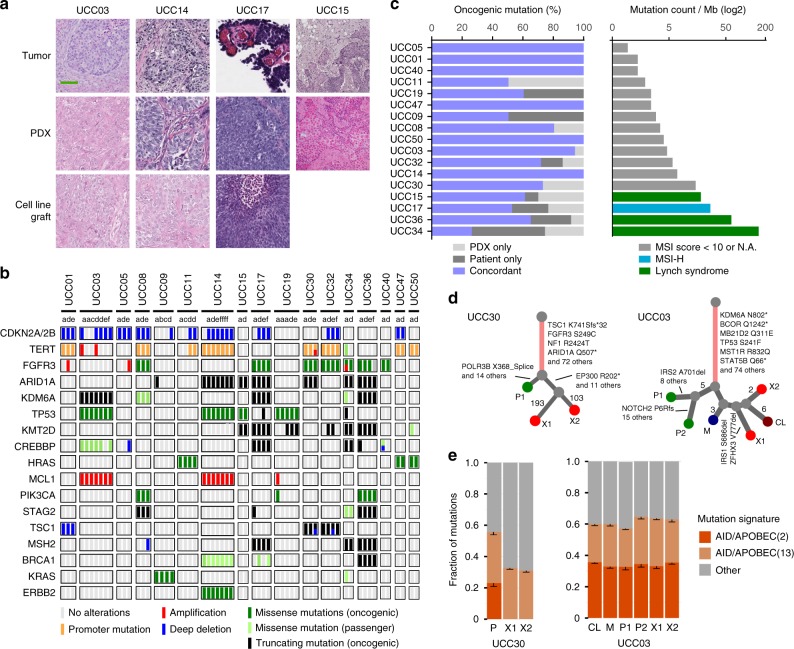
Histological and genomic comparison of patient tumors and paired patient-derived models. **a** UTUC tumors and corresponding PDX models demonstrated similar histological features as assessed by H&E staining. Scale bar corresponds to 200 µm. H&E slides of a section of each patient tumor (UCC03, *n* = 3; UCC14, *n* = 1; UCC17, *n* = 1; UCC15, *n* = 1), matching PDX (UCC03, *n* = 3; UCC14, *n* = 2; UCC17, *n* = 2; UCC15, *n* = 1) and matching cell line/cell line-derived xenograft (UCC03, *n* = 1; UCC14, *n* = 1; UCC17, *n* = 1) were reviewed by a board-certified pathologist (H.A-A.) and representative pictures are shown. **b** Concordance of selected cancer-associated genes for the 17 tumors and paired PDX models that successfully engrafted in mice [a: primary tumor, b: lymph node metastasis, c: distal metastasis, d: PDX at early passage (P1-P3), e: PDX at late passage (P4-P6), f: cell line/cell line-derived xenograft]. The UCC03 and UCC11 PDX models were established from abdominal wall and liver resections, respectively, whereas UCC32 was from a lymph node. The UCC11 PDX was generated from the frozen cell pellet of a patient specimen. All others were established from primary tumors. **c** Concordance of somatic oncogenic mutations of the patient tumor and corresponding PDX models and tumor mutational burden (TMB) of the patient tumors. Four PDX models were derived from MSI-H tumors (right panel, green and blue). **d** Phylogenetic analysis of whole-exome sequencing data for two patients (UCC30 and UCC03) revealed evidence of linear and branched tumor evolution [P: primary, M: metastasis, PDX: X1 (early passage) and X2 (late passage) which represent replicates of different passage numbers, CL: PDC]. The number of mutations is shown between branch points. **e** AID/APOBEC mutational signatures (Signatures 2 and 13) were predominant in UCC30 and UCC03 patient tumors while AID/APOBEC(2) was lost in UCC30 PDX. The mutation signatures with FDR < 0.05 are shown. Data are presented as mean values ± SD. Source data for **b**–**e** are provided as a Source Data file.

To determine whether the PDX models reflected the histological characteristics of the patient tumors from which they were derived, hematoxylin and eosin (H&E) stained sections from each were evaluated by a board-certified pathologist with expertise in GU pathology (H.A-A.) based on the current WHO classification for urinary tract tumors^19^. This analysis demonstrated histological concordance for 16/17 tumors and their corresponding patient-matched PDX models, as well as xenografts subsequently derived from the PDC models (Fig. 2a and Supplementary Table 3). For example, squamous differentiation observed in patient tumor UCC15 was retained in the corresponding PDX model.

As the properties and composition of extracellular matrix (ECM) can influence cancer cell behavior^20^ and as optimal preclinical models would be enriched with ECM similar to the original cancer tissue, we performed histological staining with Picro Sirius red followed by two-photon microscopy-second harmonics generation imaging^21,22^ on UCC36 and Masson’s trichrome staining^23^, which highlights the fibrous collagenous tracts in blue on UCC36, 17 and 19. Comparison of the collagen distribution in the tumor microenvironment of patient tumors and the corresponding patient-matched PDX models showed that both PDX and xenografts derived from PDC retained collagen structure although the percentage area covered by collagen matrix in PDX models was reduced as compared to that of the corresponding patient tumors (Supplementary Fig. 3a, b). All three PDX models and two PDC xenografts also retained the structure of matching patient tumors based on the presence of a fibrotic capsule and the presence of collagen fibers within the body of the tumor as assessed by Masson’s trichrome staining (Supplementary Fig. 3c).

To confirm that the PDX and PDC models mirrored the genomic alterations present in the tumors from which they were derived, we performed targeted sequencing using the MSK-IMPACT assay for all 17 tumor/PDX pairs that successfully engrafted. Overall, the somatic mutational profiles of the patient tumors and resulting PDX models were highly concordant (72.7% concordance of known and likely oncogenic mutations). The top recurring mutated genes found in the PDX models were *TERT* (53%), *FGFR3* (59%), *KDM6A* (24%) and *TP53* (29%) (Fig. 2b). In 29% of the PDX, we observed PDX-specific deep deletions in *CDKN2A/CDKN2B*, consistent with selection for loss of these tumor suppressor genes during the process of PDX engraftment^24^.

Four of the 17 tumors that engrafted were derived from patients with microsatellite instability high (MSI-H, MSIsensor scores ≥10) tumors including three from patients with Lynch Syndrome (germline mutations were present in *MLH1* in UCC15 and *MSH2* in UCC36, UCC34). UCC17 had loss of MSH2 and MSH6 expression by immunohistochemistry in the absence of germline mutations in either gene. One additional Lynch case failed to engraft. As would be expected, all four MSI-H tumors had a high tumor mutational burden (range: 20.3–157.2 mutation per Mb, Fig. 2c). Mutation signature decomposition analysis^25^ for all four MSI-H tumor/PDX pairs revealed stable mutational signatures across passages with the MMR/MSI and aging signatures being most predominant (Supplementary Fig. 4). Finally, the *FGFR3* R248C hotspot mutation, which has previously been shown to be enriched in Lynch Syndrome-associated UTUC as compared to sporadic UTUC tumors^26^, was present in 3 of the 4 MSI-H tumor/patient-matched PDX pairs (UCC17, UCC36 and UCC34). In sum, there was a high level of genomic concordance between radical nephroureterectomy-derived tumor samples and the corresponding PDX models (Fig. 2c) with only one PDX model, the hypermutated UCC34 PDX, displaying <50% concordance of known or likely pathogenic mutations.

To assess the extent of genomic drift resulting from establishment of the PDX and PDC models, we performed whole-exome sequencing (WES) on two cases (UCC03 and UCC30). Phylogenetic analysis of the WES data provided evidence of both linear and branched evolution in the tumors and PDX/patient-derived cell lines, as demonstrated by the presence of both private and shared mutations among samples derived from the same patient (Fig. 2d and Supplementary Fig. 5a). Analysis of the UCC03 samples, which were derived from resection of an abdominal wall metastasis, demonstrated that both the cell line (CL) and PDX (X1, X2) were more genomically related to the metastasis than to the primary tumors (P1, P2). Mutational signature decomposition analysis^26^ of the UCC03 samples identified a predominant AID/APOBEC gene editing signature (Signatures 2 and 13), a common finding in bladder UC^27,28^, in both the tumors and the PDX models. A similar AID/APOBEC gene editing signature (Signatures 2 and 13) was identified in the tumor of patient UCC30, however, the AID/APOBEC(2) signature was lost in the corresponding PDX (Fig. 2e and Supplementary Fig. 5b).

### Genomic and transcriptomic predictors of engraftment

**Fig. 3:**
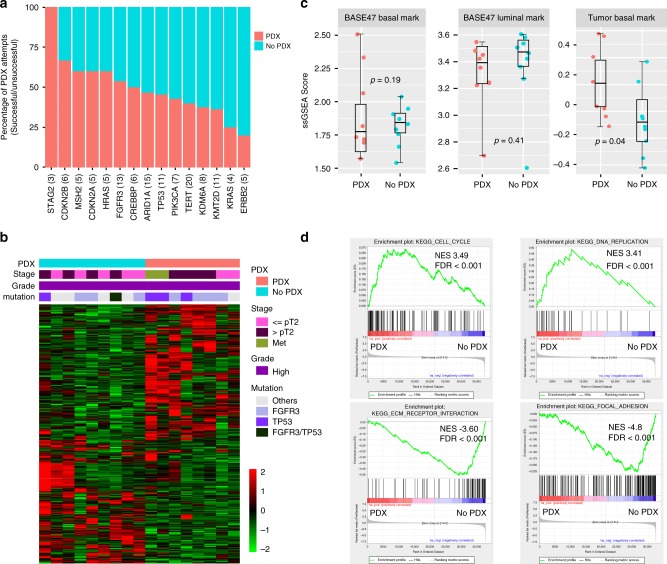
Comparison of mutation frequencies and RNA expression profiles. Patient specimens that resulted in successful engraftment (PDX) vs those that did not (No PDX) were compared. **a** The rates of successful engraftment differed among tumors with putative driver mutations. The number of patient cases with mutation in the gene is shown in *parenthesis*. **b** Comparison of RNA-seq data from patient specimens that successfully engrafted (PDX, *n* = 8, salmon) vs those that did not (No PDX, *n* = 9, turquoise). 749 genes (428 upregulated and 321 downregulated) were differentially expressed between UTUC tumors that did and did not engraft based on the *z*-score of normalized gene reads from RNA-seq. **c** Single Sample Gene Set Enrichment Analysis (ssGSEA) scores of basal (*P* = 0.19) and luminal gene sets (*P* = 0.41) based on the BASE47 classifier and ssGSEA scores of tumor basal genes (expressed in cancer cells of a basal-like phenotype, indicated at the bottom of Fig. 1b, *P* = 0.04) plotted for the patient tumors resulting in PDX (*n* = 8) vs No PDX (*n* = 9). Error bars are mean standard error from bootstrap. The center line in the boxplots indicates the mean, the lower and upper hinges correspond to the first and third quartiles, the upper whisker is the maxima and the lower whisker the minima. *P*-value indicates two-sided two group *t*-test without adjustment. **d** GSEA plots comparing specimen that did and did not yield PDX. Enrichment of cell cycle/DNA replication pathways was observed in the PDX group, whereas extracellular matrix receptor interaction and focal-adhesion pathways were enriched in the No PDX group. Source data for **b**–**d** are provided as a Source Data file.

As only 50% of the UTUC tumors successfully propagated as PDX models, we sought to determine whether successful engraftment was associated with specific mutational or transcriptomic signatures. Among the top 15 most frequently altered genes in our PDX cohort, *STAG2* and *ERBB2* mutations were positively and negatively associated with successful engraftment respectively, although the differences were not statistically significant, possibly due to the small sample size (Fig. 3a).

We next compared the transcriptomic profiles of the patient tumors that successfully engrafted to those that did not. In total, RNA-seq data was available for 17 of the UTUC patient tumors for which we attempted to establish PDX models, 8 of which engrafted and 9 of which failed to grow as PDX. There was a distinct gene expression pattern with 749 genes differentially expressed (FDR < 0.05 and Log FC > 1.5) between UTUC tumors that engrafted vs those that did not (Fig. 3b). Clustering of the patient tumors that engrafted (PDX group) vs those that failed to engraft (No PDX group) did not strongly correlate with luminal or basal classification (Fig. 1a, b) based on the BASE47 classifier^15^. More specifically, while the PDX group had higher expression of the basal gene set as compared to the No PDX group (Fig. 3c), this difference was not statistically significant. There was also no difference in ssGSEA scores for luminal genes between the PDX and No PDX groups. However, there was correlation between successful engraftment and the basal/squamous signature (*P* = 0.04) based on the consensus clustering classification^16^. In addition, GSEA revealed that genes in cell cycle and DNA replication pathways including *SMC1* (Structural maintenance of chromosomes protein 1 A), *MCM5* (DNA replication licensing factor MCM5) and *MCM7* (DNA replication licensing factor MCM7) were positively enriched in the patient tumors that successfully engrafted, suggesting that the rate of cell proliferation is an important factor for successful PDX establishment. Extracellular Matrix (ECM) receptor interaction and focal-adhesion pathways including *FN1* (Fibronectin), *THBS2* (Thrombospondin-2) and *COMP* (Cartilage oligomeric matrix protein) were also positively enriched in primary tumors that failed to produce PDX tumors, suggesting that greater dependence of tumors on microenvironmental factors may have impeded successful engraftment in a subset of patients (Fig. 3d).

### Chemosensitivity of patients and corresponding PDX

**Fig. 4:**
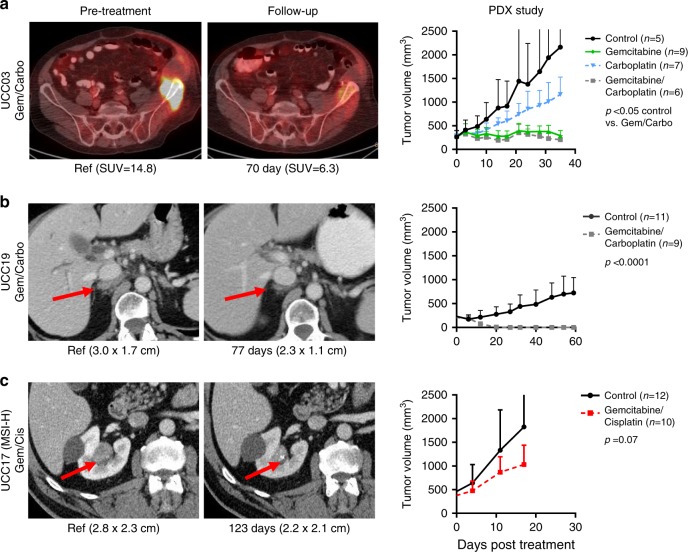
Chemosensitivity of patients and corresponding PDX. Imaging of target lesions in patients before chemotherapy (pre-treatment) and at the time specified post treatment. Tumor volumes for the corresponding PDX models were graphed as a function of days post-start of drug treatment. **a** UCC03: FDG PET/CT scan after two cycles of gemcitabine/carboplatin (Gem/Carbo) chemotherapy demonstrated decreased radiopharmaceutical accumulation in left iliac metastasis (partial response according to RECIST). Mice bearing the UCC03 PDX were randomized and treated with Gem/Carbo, gemcitabine, carboplatin or vehicle (*P* = 0.0177, vehicle vs Gem/Carbo combination; *P* = 0.0065, vehicle vs gemcitabine; *P* = 0.138, vehicle vs carboplatin). **b** UCC19 received a combination of Gem/Carbo at recurrence followed by gemcitabine alone and demonstrates stable disease by RECIST criteria off treatment for 15 months. The corresponding PDX model was highly sensitive to Gem/Carbo (*P* < 0.0001). **c** UCC17 (MSI-H) received 4 cycles of neoadjuvant gemcitabine/cisplatin. The patient’s radiographic response was minimal (stable disease by RECIST criteria) and there was no pathological response (pT3 disease at RNU). Minimal tumor growth inhibition was observed with Gemcitabine/Cisplatin in the corresponding PDX (*P* = 0.07). Two-way ANOVA test (Prism) was used for statistical analysis without adjustment. Data are presented as mean values ± SD (standard deviation). Source data for preclinical studies are provided as a Source Data file.

To evaluate whether the sensitivity of the PDX models to cytotoxic chemotherapy mirrored that of the UTUC tumors from which they were derived, mice bearing established PDX tumors from three patients were treated with chemotherapy regimens and dosing schedules similar to that of the corresponding patients. As an example, patient UCC03 developed abdominal wall, bone and pelvic metastases 11 months after RNU for pT3bN0 disease. The patient was treated with palliative excision of the left abdominal wall metastasis and radiotherapy followed by gemcitabine/carboplatin (Gem/Carbo) chemotherapy. A PET-CT at 3-months follow-up demonstrated a partial response by RECIST criteria as well as decreased ^18^F-fluorodeoxyglucose (FDG) avidity within the dominant pelvic osseous metastasis. A PDX derived from the abdominal metastasis was similarly responsive to Gem/Carbo (Fig. 4a).

Patient UCC19 underwent RNU for pT3 disease and relapsed 16 months later with retroperitoneal lymphadenopathy. The patient responded to Gem/Carbo followed by gemcitabine maintenance and has been off therapy with RECIST stable disease for the past 15 months. Consistent with the patient’s durable response to chemotherapy, a PDX generated at the time of RNU (UCC19) demonstrated exquisite sensitivity to Gem/Carbo (Fig. 4b). As DNA damage response (DDR) gene alterations have been associated with sensitivity to platinum-based chemotherapy and improved survival in patients with advanced UC patients^29,30^, we assessed whether there was evidence of a likely pathogenic DDR gene alteration. Indeed, both the primary tumor and corresponding PDX harbored a truncating mutation in *RECQL4* (F987*), a gene which has a role in DNA repair by homologous recombination^31^.

Patient UCC17, who had an MSI-H tumor, received gemcitabine/cisplatin neoadjuvant chemotherapy (NAC) to which he demonstrated minimal decrease in size on CT from 2.8 to 2.2 cm (stable disease by RECIST criteria) (Fig. 4c) and no pathologic response (pT3N0 at RNU). The PDX generated from the primary tumor was also unresponsive to gemcitabine/cisplatin (*P* = 0.07). Similarly, UCC36 which was derived from a patient with an MSI-H Lynch syndrome-associated UTUC was also resistant to gemcitabine/cisplatin (Supplementary Fig. 6). As chemoresistance has been reported in colorectal cancer patients with MSI-H tumors^32,33^ and as the MSI-H UCC17 and UCC36 models were relatively resistant to gemcitabine/cisplatin chemotherapy, we assessed MSI status and chemotherapy responsiveness in a surgical cohort of UTUC patients treated with NAC. Of the 20 patients with UTUC who received NAC before RNU, two were MSI-H (one of which was UCC17), one was MSI-indeterminant (MSIsensor scores 3–10), and 17 were microsatellite stable (MSS, MSIsensor scores <3). Defining pathologic response as ≤pT1N0^34,35^, we observed pathologic response in 10 of 17 MSS patients (59%) and in neither of the two MSI-H patients.

### HER2-targeted therapy in HER2-mutant UTUC

**Fig. 5:**
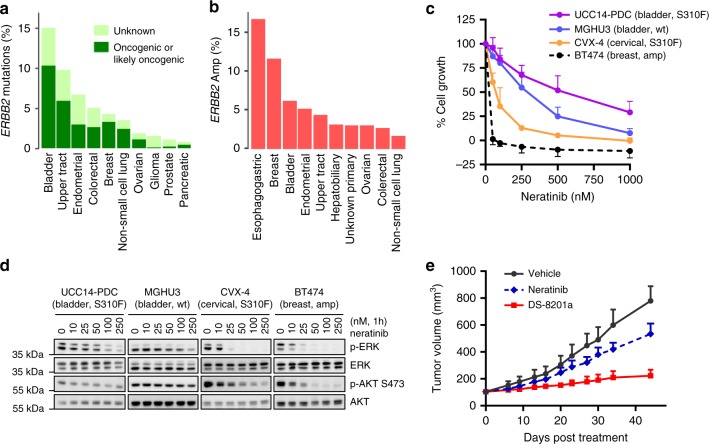
Assessment of HER2-targeted strategies. **a**, **b** Fraction of patients with *ERBB2* mutation or amplification, respectively, in bladder, UTUC and other common solid tumors in a prospectively sequenced cohort of 44,183 tumors. **c** Neratinib sensitivity of the UCC14-PDC (HER2 S310F-mutant UTUC), MGHU3 [*ERBB2* wildtype (wt) bladder cancer], CVX-4 (HER2 S310F-mutant cervical cancer) and BT-474 (*ERBB2* amplified breast cancer) was determined 5 days post-neratinib treatment. The average values from three separate experiments (*n* = 3) for UCC14-PDC, MGHU3, and CVX-4 and 5 separate experiments (*n* = 5) for BT-474 are shown. IC50s for neratinib were 508.3, 245.9, 56.8 and 0.1 nM for UCC14, MGHU3, CVX-4 and BT-474, respectively. **d** Inhibition of ERK and AKT, downstream HER2 effectors by neratinib. Western blot was performed (repeated three times) on samples treated with neratinib or vehicle for 1 h at the indicated concentrations. The samples were derived from the same experiment and the gels/blots were processed in parallel. **e** Mice with established UCC14 tumors were treated with neratinib 20 mg/kg PO daily (5 days a week), DS-8201a 10 mg/kg i.v. once every 3 weeks or vehicle only as control. Mice bearing the PDX were randomly assigned to 3 cohorts (*n* = 8 per each group) and monitored twice a week [*P* = 0.0034, vehicle vs neratinib; *P* < 0.0001, vehicle vs DS-8201a; *P* < 0.0001, neratinib vs DS-8201a]. Two-way ANOVA test (Prism) was used for statistical analysis. Data are presented as mean values ± SD. Source data for **c**–**e** are provided as a Source Data file.

Consistent with retrospective studies, prospective genomic profiling of 44,183 tumors including 1032 bladder and 204 UTUC at our institution using MSK-IMPACT revealed that mutations in *ERBB2* (oncogenic/likely oncogenic) were more common in urothelial cancer (UC) than in other common solid tumors (10.3% in bladder and 5.9 % in UTUC as compared to 3.2% in breast, 2.9% in endometrial, 2.6% in colorectal, and 2.4% in NSCLC) (Fig. 5a). *ERBB2* amplification was also common in bladder cancer and UTUC (6.2%: 64 of 1032 bladder tumors and 4.4% of UTUC: 9 of 204 tumors, Fig. 5b). As HER2 is a validated drug target in breast and esophagogastric cancers, there has been enthusiasm for targeting HER2 in patients with UC. However, only limited efficacy has been observed to date with HER2 kinase inhibitors, such as an irreversible EGFR/HER2 kinase inhibitor, neratinib^36^.

Efforts to define the dependence of HER2-mutant urothelial carcinoma cells on HER2 signaling have been hampered by the lack of available preclinical models of HER2-mutant UC. As genomic analysis revealed that the UCC14 patient tumor and paired PDX harbored a hotspot mutation in *ERBB2* (HER2 S310F), we sought to leverage this model to explore the biological basis for the limited anti-tumor activity of HER2-targeted therapies in patients with UC. We thus generated a cell line from an early passage UCC14-PDX (UCC14-PDC) and assessed its sensitivity to neratinib, a HER2 kinase inhibitor. Both UCC14-PDC (IC50 508.3 nM) and MGHU3 (an *ERBB2* wildtype bladder cancer line, IC50 245.9 nM) were significantly less sensitive to neratinib as compared to CVX-4^37^ (a HER2 S310F-mutant cervical cancer line, IC50 56.8 nM) and BT-474 (an *ERBB2* amplified breast cancer cell line, IC50 0.1 nM)(Fig. 5c). The greater concentration of neratinib required to inhibit UCC14-PDC cell growth as compared to other HER2 altered lines was consistent with the higher concentration of drug needed to inhibit activation of ERK, a downstream effector of HER2 (Fig. 5d). Expression of phosphorylated AKT was also potently downregulated by neratinib in BT-474 and CVX-4 cells, but was unchanged in UCC14-PDC, suggesting that AKT pathway activation was not HER2 dependent in this UC model. Finally, the limited sensitivity of the HER2 S310F-mutant UCC14-PDC to HER kinase inhibition in culture was consistent with the relative insensitivity of the UCC14-PDX to neratinib therapy in vivo (Fig. 5e). In contrast to the lack of efficacy of neratinib, DS-8201a, a humanized HER2 antibody topoisomerase I inhibitor drug conjugate (trastuzumab deruxtecan, Daiichi-Sankyo) recently approved by the FDA for unresectable or metastatic HER2-positive breast cancer (NCT03248492)^38^, significantly inhibited UCC14-PDX growth. These data suggest that antibody drug conjugates targeting HER2 may be more effective than selective HER kinase inhibitors in patients with HER2-mutant UC.

## Discussion

A major hurdle to the development of personalized treatment approaches for UTUC has been the lack of laboratory models for biological and preclinical studies. Here, we report the development and biological and genomic characterization of PDX and PDC models from patients with UTUC. We find that the PDX and PDC models recapitulated the diverse landscape of genomic alterations in UTUC. Furthermore, in the select cases tested, the PDX models mirrored the sensitivity/resistance to standard cytotoxic agents of the patients from which the PDX models were derived.

Transcriptomic-based clustering analysis of 80 UTUC tumors using the BASE47 gene classifier^15^ found that the majority (70/80, 87.5%) of tumors had a luminal phenotype. Further, application of the recently reported Bladder Cancer Molecular Taxonomy Group consensus classifier resulted in 66 of 80 tumors being classified as luminal–papillary sub-type, including all 14 tumors with low-grade histology (Fig. 1b)^16^. These data are consistent with those of Robinson et al.^5^ and suggest that the luminal–papillary sub-type is enriched in UTUC as compared to bladder cancer where only 24% of tumors were luminal–papillary sub-type^16^.

Another notable difference between urothelial carcinomas arising in the upper tract and the bladder is the significantly higher incidence of microsatellite instability in renal pelvis and ureteral tumors. It has been reported that patients with Lynch syndrome-associated colorectal cancer have diminished response to chemotherapy relative to patients with sporadic colorectal cancer^32^. Our data suggest that a similar association between chemotherapy sensitivity and MSI status might exist in patients with UTUC. More specifically, we evaluated the sensitivity of two UTUC PDX models (UCC17 and UCC36) derived from patients with MSI-H tumors to platinum chemotherapies and gemcitabine and found that both were relatively resistant to these cytotoxic chemotherapies. Given recent studies suggesting that MSI may be a predictive biomarker of response to immune checkpoint blockade in multiple cancers^39,40^, these results suggest that MSI status may be useful in assessing whether UTUC patients should receive neoadjuvant cytotoxic chemotherapy, or, instead be directed to immunotherapy-based clinical trials.

Despite the recent FDA-approval of erdafitinib for *FGFR3*-mutated metastatic bladder cancers^41^, the clinical impact of targeted therapies in UC has been limited to date. *ERBB2* is altered by amplification and/or overexpression in various cancer types, and antibodies that bind to the extracellular domain of HER2 are now standard-of-care in *ERBB2* amplified breast and esophagogastric cancers^42,43^. Our prospective clinical sequencing experience of over 44,000 patient tumors indicates that activating *ERBB2* mutations are more prevalent in both bladder and UTUC than in other common solid tumor types (Fig. 5a). However, the lack of *ERBB2* mutant models of UTUC has been a major impediment to the preclinical assessment of HER2-targeted therapies for UTUC patients.

As the UCC14-PDX and patient tumor from which it was derived harbored an activating hotspot mutation in the extracellular domain of HER2 (S310F), we leveraged this model to explore the biological basis for the limited clinical activity of HER kinase inhibitors observed to date in HER2-mutant UC patients. Consistent with the poor response of HER2-mutant UC patients to the HER kinase inhibitor neratinib as compared to HER2-mutant breast and cervical cancer patients^36^, the HER2 S310F-mutant cell line UCC14-PDC was significantly less sensitive to neratinib than the HER2-amplified BT-474 breast cancer and HER2 S310F-mutant CVX-4 cervical cancer cell lines. The relative lack of neratinib sensitivity of the UCC14 cell line was consistent with the higher doses of neratinib required to inhibit downstream ERK signaling in this cell line model as compared to BT-474 and CVX-4 cells^37^. This result suggests that the variable sensitivity of different tumor lineages to HER2-directed therapies as reported in a recent basket trial^36^, is likely due, at least in part, to differences among cancers in their dependence on HER2 signaling for tumor growth and survival.

Li et al.^44^ recently reported that the HER2 antibody drug conjugate ado-trastuzumab emtansine (T-DM1) has significant clinical activity in HER2-mutant lung cancer, even in tumors with low HER2 expression. One hypothesis as to why HER2-mutant tumors with low HER2 expression are sensitive to T-DM1 is that mutation of HER2 may increase the efficiency of receptor internalization following antibody binding^45^. Consistent with this finding, the UCC14-PDX was highly sensitive to DS-8201a, a HER2 antibody drug conjugate, which was recently approved by the FDA for unresectable or metastatic HER2-positive breast cancer^38^. In sum, the data highlight the potential ability of PDX models to guide the development of clinical trials for UTUC patients and suggest that antibody drug conjugates targeting HER2 may be more effective in UTUC patients than selective HER2 kinase inhibitors.

A limitation of the current study is that detailed biological characterization of the large number of variants of unknown significance identified in the PDX models is beyond the capability of any single laboratory. Thus, to facilitate biological discovery and the development of novel therapies for UTUC, all genomic data from the current work is available through cBioPortal [https://www.cbioportal.org/study/summary?id=utuc_msk_2019; https://www.cbioportal.org/study/summary?id=utuc_pdx_msk_2019] and all models reported here will be provided as a resource to the broader scientific community. In addition, the current collection of PDX models was mostly derived from large surgical specimens. Generating PDX models from ureteroscopic biopsies in the pre-radical nephroureterectomy setting would be of particular interest to assess the potential benefits of neoadjuvant chemotherapy for patients with UTUC. This will be the focus of future work seeking to leverage PDX models to inform clinical decision-making.

## Methods

### Specimen acquisition

From March 2014 to April 2017, tumors and matched normal tissue from 34 patients with UTUC (31 radical nephroureterectomy [RNU] specimens, 2 samples from distant metastasis and 1 UTUC biopsy) were collected under protocols (NCT01775072, MSKCC IRB #89-076 and IRB #06-107) approved by the Memorial Sloan Kettering Cancer Center (MSKCC) Institutional Review Board. We have obtained informed consent from all participants of this study. RNU specimens were dissected robotically in a usual manner with the hilum stapled as late as feasible to limit ischemia time. Extraction of the specimen was performed immediately to ensure no more than 20–30 min of surgical ischemia time before specimen removal. The specimens were then transported directly to pathology, tumor(s) was identified grossly by a pathologist and a fresh sample for PDX generation was collected under sterile condition. Surgical samples were then transported to a research laboratory in RPMI medium or PBS on ice. Time from surgical removal of tissue in the OR to placement in medium in pathology and time from pathology to the research laboratory was ~20–40 min and 10 min, respectively. For all specimens, representative hematoxylin and eosin (H&E) slides from frozen tissue and formalin-fixed paraffin-embedded (FFPE) sections were reviewed by a board-certified genitourinary pathologist (H.A-A.) to confirm histology, grade and stage. Clinical and demographic information was obtained from a prospectively maintained institutional database.

### Establishment of xenografts from surgical specimen

All animal work was approved by the MSKCC Institutional Animal Care and Use Committee (IACUC). We have complied with all relevant ethical regulations. All surgical specimens were transported in RPMI medium or PBS on ice, washed with cold PBS, cut into small pieces (2 × 2 to 3 × 3 mm^3^) and then implanted subcutaneously into one or two sides of the flank of 6–8-week-old male immunocompromised NOD-SCID *IL2Rg*^−/−^ (NSG) mice (Jackson Laboratory) via a 4–5 mm skin incision over the mid-lumbar spine^46,47^. PDX from UCC11 was generated from a frozen cell pellet after a collagen-based digestion process. PDX were serially transplanted for expansion when the tumor volume reached 0.5–1 cm^3^. Harvested xenograft tumors were cut into small fragments as earlier and either stored in freezing media (Recovery™ Cell Culture Freezing Medium, Thermo Fisher Scientific) in liquid nitrogen for future implantation and/or flash frozen for molecular studies including RNA-seq and/or western blot analysis.

### Primary culture and xenograft implantation

Tumor tissues were minced to small pieces, processed using 225 U/ml Collagenase type III at 37 °C for 15–30 min before plating into collagen-coated plates in advanced DMEM/F12 medium, supplemented with 10% FBS, 2 mM Glutamax, 1 mM sodium pyruvate, 1× non-essential amino acid, 10 mM HEPES, 100 µg/ml primocin (InVivoGen), 50 ng/ml EGF (MilliporeSigma) and 10 µM Y-27632 (Selleck Chemicals). All cell culture components were from Thermo Fisher Scientific unless otherwise stated. When the plates became confluent, cells were trypsinized using 0.25% TrypLE for expansion. For generation of the UCC14 patient-derived cell line (UCC14-PDC), a PDX piece at passage 3 was processed using 225 U/ml Collagenase type III, mixed with Matrigel (growth factor reduced; Corning Life Sciences) at 1:1 and plated as multiple 50 µl drops in 6-well plates to establish a 3D culture and then converted to a cell line for growth in 2D for in vitro assays. For in vivo tumorigenesis studies of the patient-derived cell lines shown in Supplementary Fig. 2, 3–5 million cells of PDC in media mixed with Matrigel at 1:1 were subcutaneously injected into the flank of NSG mice and tumor growth was measured weekly by calipers.

### Targeted (MSK-IMPACT) and whole-exome sequencing

Targeted sequencing was performed using the MSK-IMPACT (Memorial Sloan Kettering Integrated Mutation Profiling of Actionable Cancer Targets) assay using FFPE tumors and matched FFPE normal tissue or frozen blood. Matched normal tissue samples were obtained from renal parenchyma or lymph nodes that were confirmed to be histologically benign. MSK-IMPACT is a hybridization capture-based assay for deep sequencing of cancer-associated genes. A 300 gene version was used for the UTUC samples analyzed retrospectively, whereas 341, 410 or 468 versions were used for the prospectively sequenced tumors and PDX and PDC models. Briefly, following DNA extraction, an equimolar pool of barcoded libraries was subjected to exon capture using custom oligonucleotides, sequenced on an Illumina HiSeq 2500 instrument (Illumina Inc), and analyzed using a customized pipeline for somatic mutation calling, copy number alterations and structural rearrangements^6^.

DNA samples for whole-exome sequencing were barcoded and then processed using SureSelect Human All Exon V4 (Agilent Technologies) according to the manufacturer’s instructions followed by sequencing on a HiSeq 2500 instrument in 100-bp paired-end mode. The average aligned reads per samples were 95 million, with 235-fold coverage on targeted regions, and an average duplication rate of 17.6%. For phylogenetic analysis, sample distances between pairs of samples were calculated based on non-shared mutations, followed by hierarchical clustering using the average agglomeration method. We used the Stringlist package in R software to count the non-shared mutations and the Analysis of Phylogenetics and Evolution package to plot the tree. The resulting phylogenetic trees were then denoted with notable oncogenic mutations.

### RNA sequencing and transcriptome analyses

Total RNA from frozen tissue was isolated using Trizol (Thermo Fisher Scientific) and the quantity and quality of the resulting RNA was measured using an Agilent 2100 Bioanalyzer. The TruSeq RNA Library Prep Kit v2 (Illumina) was used for library preparation, followed by sequencing (60 million paired-end reads) on an Illumina HiSeq 2500. Read numbers for genes were extracted by RSEM (using the STAR alignment program)^48^. Mean aligned reads per sample was 25 million. The consensusMIBC package (https://github.com/cit-bioinfo/consensusMIBC, Bladder Cancer Molecular Taxonomy Group)^16^ was used for a consensus clustering analysis. Differentially expressed genes between the tumors that led to PDX (PDX group) vs those that failed to produce PDX (No PDX group) were identified by DESeq2^49^. Gene Set Enrichment Analysis was applied to identify pathways significantly different in the PDX vs No PDX groups^50^.

### Two-photon microscopy and second harmonics generation

Unstained patient, PDX and PDC-derived FFPE tumors were cut into 15-μm-thick sections, de-paraffinized, hydrated and visualized using a two-photon microscope (2PM: Zeiss LSM 880 upright Laser Scanning Microscope) with non-linear optics, coupled with a Chameleon MPX (Coherent Inc.) femtosecond pulsed, tunable Ti: Sapphire laser for two-photon excitation with Plan Apochromat ×20/0.8 (Carl Zeiss, Germany). For collagen second harmonic imaging, a wavelength of 880 nm was used. Both reflected and transmitted signals were collected for image analysis and processed using Zeiss Zen imaging software.

### Picro Sirius red staining

The patient, PDX and PDC-derived tumor FFPE sections were stained with Picro Sirius red^51^ to quantitate changes in deposition or architecture of extracellular matrix structures, specifically collagen fibers. De-waxed and hydrated paraffin sections were stained for 1 h and washed in two changes of acidified water. Slides were dehydrated in three changes of 100% ethanol, cleared in xylene and mounted in a resinous medium. Slides were scanned using Pannoramic scanner (3D Histech Ltd.) and analyzed using Pannoramic viewer (3D Histech Ltd.) and FIJI (Image J) software.

### Masson’s trichrome staining

For direct visualization of collagen fibers and histological assessment of collagen deposition, patient, PDX and PDC-derived tumor FFPE sections were de-waxed, hydrated and a trichrome staining^23^ was performed using the Masson Trichrome Staining Kit (Sigma-Aldrich, St Louis, MO, USA). Slides were dehydrated quickly in 95% and absolute ethanol, cleared in xylene and mounted. Slides were scanned using Pannoramic scanner (3D Histech Ltd.) and analyzed using Pannoramic viewer (3D Histech Ltd.).

### Treatment of PDX-bearing mice with chemotherapy

Chemotherapy was administered intraperitoneally when tumor volumes reached 100–300 mm^3^. For drug studies, mice were randomly assigned into vehicle control (saline for gemcitabine and platin drugs) and treatment groups (6–12 mice per each group). Mice were treated with the combination of weekly 50 mg/kg of gemcitabine and 5 mg/kg cisplatin (4-week cycle; weekly gemcitabine with 4th week off and cisplatin at every 4 weeks) or the combination of weekly 50 mg/kg of gemcitabine and 50 mg/kg carboplatin (4-week cycle). Tumor growth was quantitated weekly using calipers and growth curves were generated using GraphPad Prism software. Following guidelines set forth by the IACUC, mice were killed when tumor volume reached 1 cm^3^. All drugs were purchased from Selleck Chemicals.

### Neratinib and DS-8201a sensitivity assays

UCC14-PDC (*ERBB2* S310F mutated bladder cancer), BT-474 (*ERBB2* amplified breast cancer), MGHU3 (*ERBB2* wildtype bladder cancer, kindly provided by Dr. Margaret Knowles) and CVX-4 (*ERBB2* S310F mutated cervical cancer) cells were treated with neratinib (Puma Biotechnology) at concentrations ranging from 0 to 1000 nM. For growth assays, cells were seeded into 6-well plates. After allowing 24 h for adherence, cells were then counted at Day 0 time point, or at Day 5 post treatment with neratinib or DMSO using the Vi-CELL XR 2.03 (Beckman Coulter). Growth curves were generated from the averages of at least 3 experiments in duplicate (±SE) by plotting percent growth [(mean cell number at drug dose D5 − mean cell number of control D0)/(mean cell number of DMSO D5 − mean cell number of control D0) × 100)] against drug concentration. For in vivo drug studies, a UCC14-PDX tumor was mechanically dissociated, resuspended in Matrigel at 1:1 and subcutaneously injected into the flanks of male NSG mice (Jackson Laboratories). Once tumors reached an average volume of 100 mm^3^, mice were randomized to receive either vehicle control (0.5% methylcellulose/0.2% Tween 80 for neratinib, PBS for DS-8201a), neratinib, or DS-8201a (Daiichi-Sankyo). The dosing schedules were as follows: neratinib at 20 mg/kg p.o. daily 5 days/week, DS-8201a at 10 mg/kg i.v. once every 3 weeks. Tumors were measured twice a week using calipers. Interested parties should contact Dr. Alessandro Santin for CVX-4 and Dr. Margaret Knowles for MGHU3 cell lines.

For western blot studies, cells were seeded into 60 mm dishes. At 75% confluence in log phase growth, cells were treated with neratinib at 0–250 nM for 1 h and then lysed in 1% NP-40 lysis buffer or RIPA buffer and processed for immunoblotting^37,52^. For primary antibodies, phospho-antibodies were used at 1:500 in TBST/5%BSA and totals were used at 1:1000 TBST/5% BSA with incubation overnight at 4 °C. Secondary antibodies were used at 1:2000 TBST/5% BSA for 1 hr at room temperature. ERK (#9102), p-ERK (T202/Y204) (#9101), AKT (#9272) and p-AKT (Ser473) (#9271) antibodies were from Cell Signaling Technology. Proteins were visualized using the Fuji LAS-4000 (GE LifeSciences).

### Statistical analysis

A xenograft was deemed to have successfully engrafted when the tumor volume reached 1 cm^3^ or the maximum tumor diameter reached 1.5 cm, and urothelial carcinoma was confirmed by histopathology of the grafted tumors. Clinical variables of patients and differences in engraftment rates were analyzed using Fisher’s exact testing for categorical variables and Wilcoxon rank sum for continuous variables. Treatment effects on tumor growth were evaluated by two-way analysis of variance (two-way ANOVA, GraphPad Prism software Version 8). R/Bioconductor packages^53^ were used to analyze RNA-Seq data (DESeq2 package) and DNA mutations (maftools package). In addition, gene set variation analysis was performed using single sample GSEA (within GSVA package).

### Reporting summary

Further information on research design is available in the Nature Research Reporting Summary linked to this article.

## Supplementary information

Supplementary Information

Reporting Summary

## Data Availability

All genomic data are accessible on cBioPortal at the following two links; Upper Tract Urothelial Carcinoma (MSK, 2019) [https://www.cbioportal.org/study/summary?id=utuc_msk_2019] or Upper Tract Urothelial Carcinoma PDX (MSK, 2019) [https://www.cbioportal.org/study/summary?id=utuc_pdx_msk_2019]. The raw and normalized RNA sequencing data are available in the database of Gene Expression Omnibus (GEO) associated with series entry number GSE134292. The source data underlying Figs. 1a, b, 2b–e, 3b–d, 4a–c, 5c–e and Supplementary Figs. 2, 4, 6 are provided as a Source Data File.

## Source Data

Source Data
