# Supplementary material for: Modeling biological and genetic diversity in upper tract urothelial carcinoma with patient derived xenografts
Source: Nat Commun. 2020 Apr 24;11:1975. doi: 10.1038/s41467-020-15885-7 (PMC7181640; doi:10.1038/s41467-020-15885-7)
Supplement: Supplementary file 1 — Supplementary Information [file 41467_2020_15885_MOESM1_ESM.pdf]

Supplementary information to:

Modeling Biological and Genetic Diversity in Upper Tract Urothelial Carcinoma with Patient Derived Xenografts

By K Kim, W Hu et al.

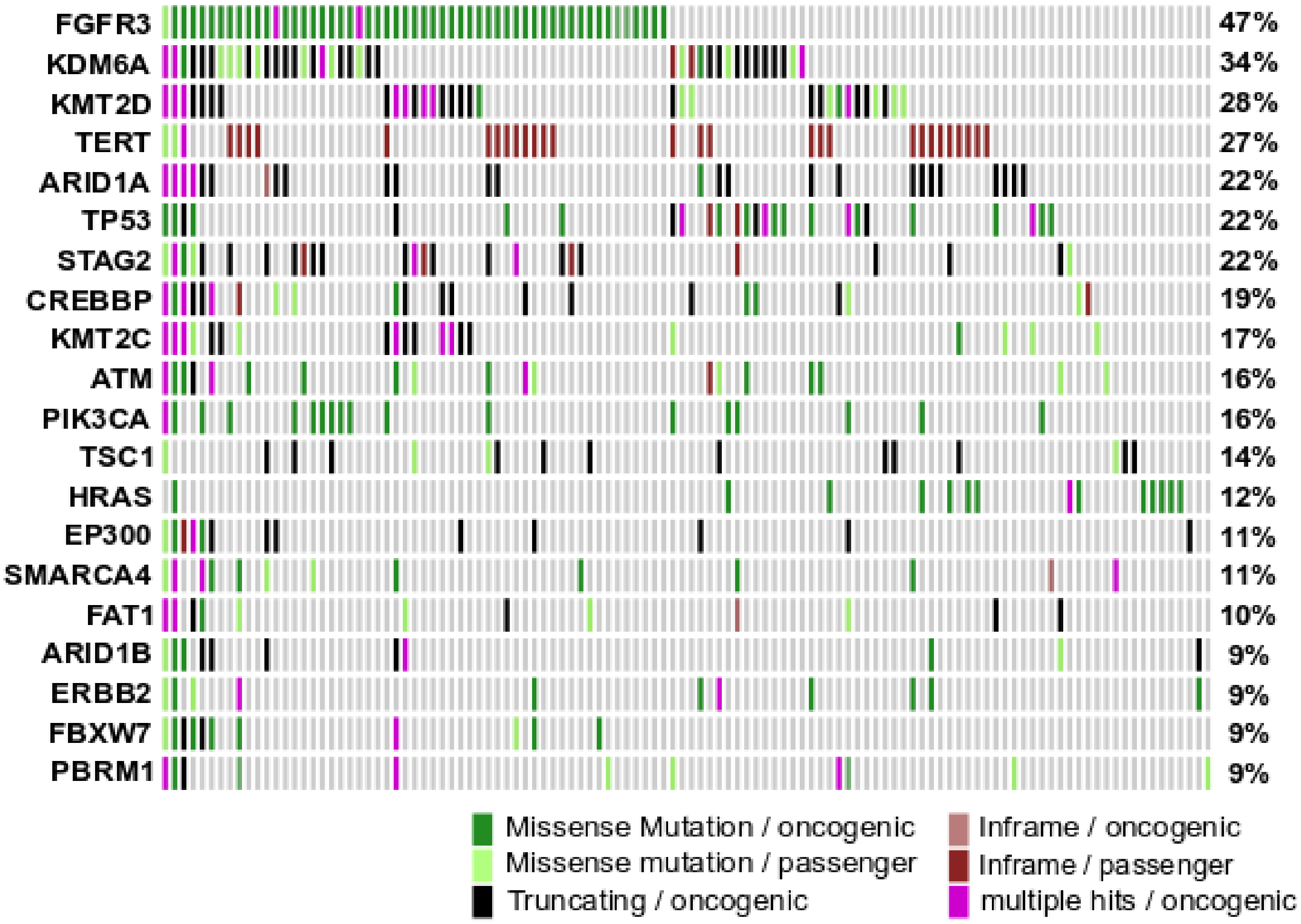

Supplementary Fig. 1 Genomic analysis of 119 prospectively and retrospectively sequenced UTUC tumors using MSK-IMPACT. An oncoprint of the 20 most frequently mutated genes is shown.

Supplementary Table 1 Clinico-pathological characteristics of the UTUC samples analyzed by RNA-seq.

Characteristic	Number of patients (%)
Number of patients	80
Male gender	53 (66%)
Race	
Caucasian	73 (91%)
African-American	2 (2.5%)
Asian	2 (2.5%)
Hispanic	1 (1.5%)
Unknown	2 (2.5%)
Smoking status	
Never	20 (25%)
Former	49 (61%)
Active	11 (14%)
Histology	
UC, NOS	75 (94%)
UC with variant histology	5 (6%)
High grade	66 (83%)
Pathologic tumor stage	
pTa	23 (29%)
pT1	14 (17%)
pT2	11 (14%)
pT3	27 (34%)
pT4	5 (6%)
Pathologic node stage	
pN0	53 (66%)
pN1-2	15 (19%)
pNx	12 (15%)
Positive surgical margin	5 (6%)
Received NAC	6 (8%)
Received adjuvant chemotherapy	3 (4%)
Disease recurrence	35 (44%)

Supplementary Table 2 Propagation and preservation of PDX models of UTUC. To minimize the risk of a loss of tumorigenicity with subsequent passages, multiple early-passage tumor fragments were frozen for future implantation. The viability of freeze-thaw has been confirmed for majority of cases before stop propagation except UCC15 line failing for engraftment after freeze/thaw process.

UTUC sample	Highest Passage	Viability checked post freeze/thaw	frozen stocks
UCC01	P6	Yes at P3	multiple passages
UCC03	P7	Yes at P2	multiple passages
UCC05	P6	Yes at P2	multiple passages
UCC08	P4	Yes at P3	multiple passages
UCC09	P5	Yes at P4	multiple passages
UCC11	P6	NA	multiple passages
UCC14	P9	Yes at P4	multiple passages
UCC15	P4	NA	Line is lost at P4 after freeze/thaw
UCC17	P9	Yes at P1, P2, P5	multiple passages
UCC19	P5	Yes at P4	multiple passages
UCC30	P7	Yes at P2	multiple passages
UCC32	P7	Yes at P3	multiple passages
UCC34	P3	NA	multiple passages
UCC36	P5	Yes at P3	multiple passages
UCC40	P7	NA	multiple passages
UCC47	P6	NA	multiple passages
UCC50	P4	NA	multiple passages

Line ID	Origin	Passage	In vivo growth
UCC03	PDX from abdominal metastasis	> P15	Yes
UCC14	PDX from primary	> P15	Yes
UCC17	Primary	> P15	Yes
UCC32	Primary	> P15	No
UCC36	Primary	> P11	Yes
UCC47	Primary	> P15	Not determined

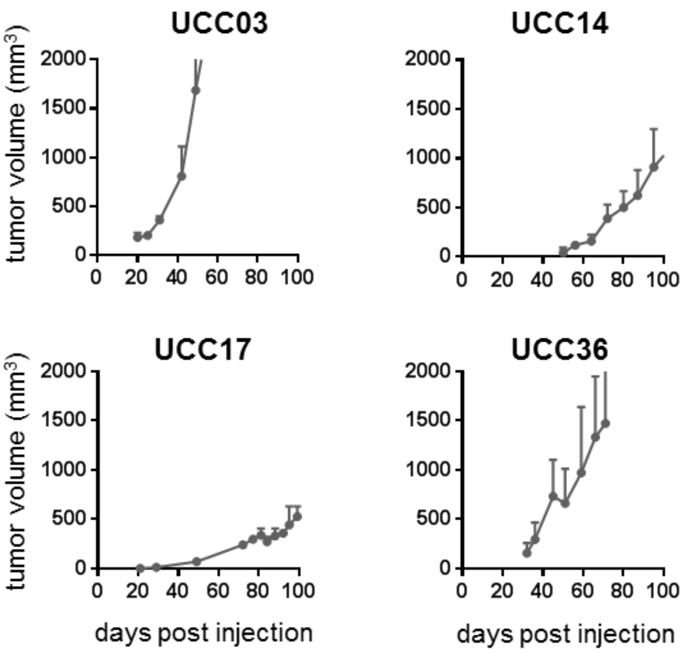

Supplementary Fig. 2 *In vivo* tumorigenicity of PDC. Four PDC were directly generated from patient specimens while two PDC (UCC03, UCC14) were derived from PDX tumors at passage 3. Four of 5 PDC tested were able to form tumors *in vivo* following subcutaneous injection of cells into NSG mice. PDC; patient-derived cell line, PDX; patient-derived xenografts. Data are presented as mean values +/- SD.

Supplementary Table 3. Pathology review of patient-matched UTUC tumor and PDX models. Sixteen of 17 cases were concordant on histologic review with the exception being UCC34. NOS; not otherwise specified.

UTUC sample	patient tumor	matching PDX tumor
UCC01	UC, NOS	UC, NOS
UCC03	UC, NOS	UC, NOS
UCC05	UC, NOS	UC, NOS
UCC08	UC, NOS	UC, NOS
UCC09	UC with nested and microcystic changes	UC with nested and microcystic changes
UCC11	UC with focal squamous differentiation	UC with focal squamous differentiation
UCC14	UC, NOS	UC, NOS
UCC15	UC exhibiting a predominant nested pattern of growth and focal squamous differentiation	UC exhibiting a predominant nested pattern of growth and focal squamous differentiation
UCC17	UC, NOS	UC, NOS
UCC19	UC with focal squamous differentiation	UC with focal squamous differentiation
UCC30	UC, NOS	UC, NOS
UCC32	UC, NOS	UC, NOS
UCC34	UC, NOS	UC with squamous differentiation
UCC36	UC, NOS	UC, NOS
UCC40	UC, NOS	UC, NOS
UCC47	UC, NOS	UC, NOS
UCC50	UC, NOS	UC, NOS

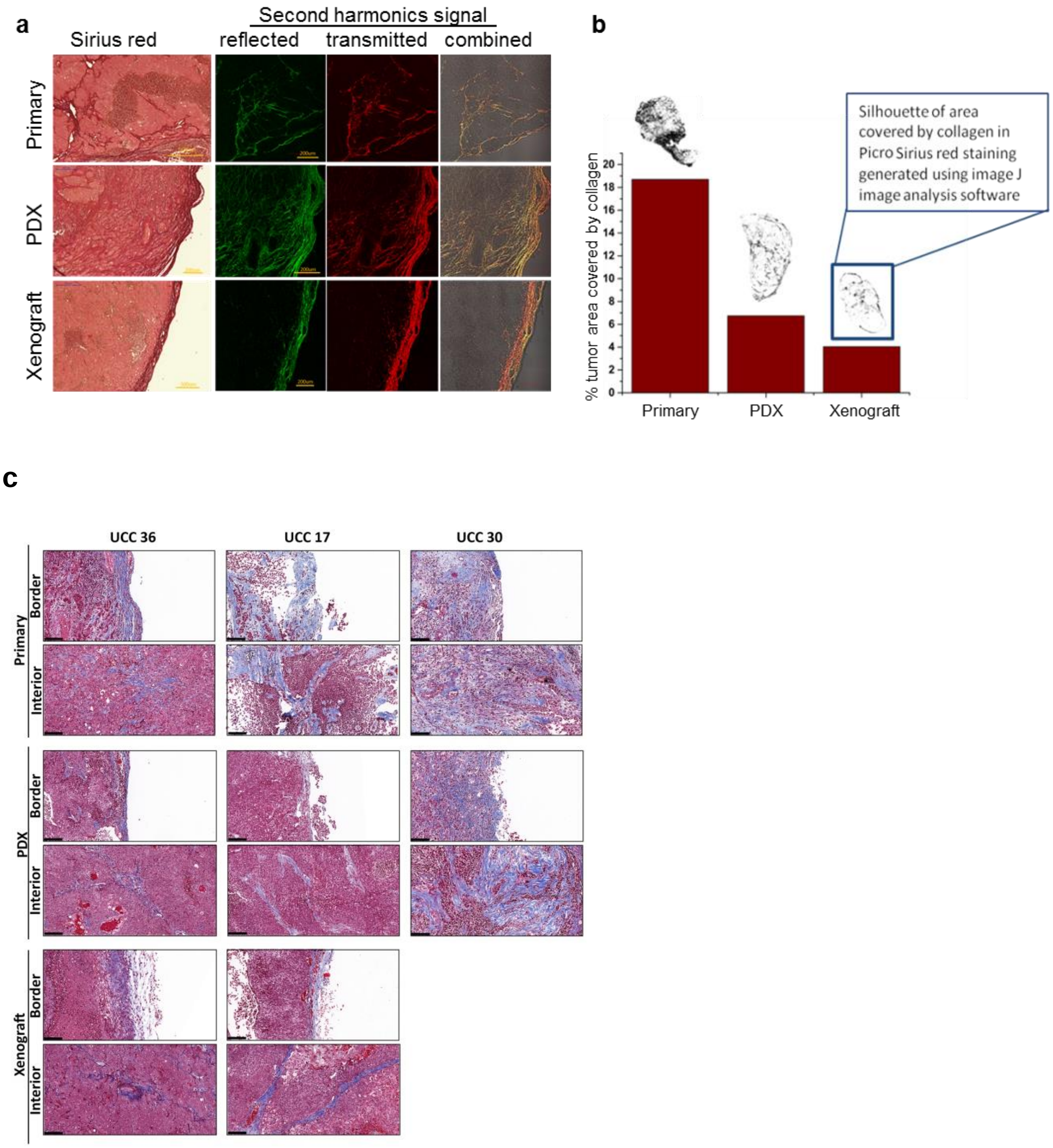

Supplementary Fig. 3 Comparative examination of extracellular matrix architecture between matched patient tumors, patient derived xenografts (PDX) or/and a xenografts generated from the corresponding patient derived cell line (PDC). **a** UCC36 tumors stained with Picro Sirius Red (left panel, n=1 per each staining) or unstained tissues were imaged using second harmonics generation with a 2 Photon microscope at 880nm (right panel, n=1 per each staining). Scale bar corresponds to 200 µm **b** Quantification of the area covered by collagen in Sirius Red staining. The collagen sheets of PDX and xenograft were similar in architecture but covered a smaller percentage area compared to the corresponding patient tumor. **c** Masson's trichrome staining which highlights the fibrous collagenous tracts in blue was performed for three sets of patient tumor, matching PDX and xenografts derived from PDC. All three of the primary tumors and their corresponding PDX or PDC xenografts examined exhibited the presence of a well-defined border and presence of fibrous structures inside the tumor. Scale bar corresponds to 100 µm.

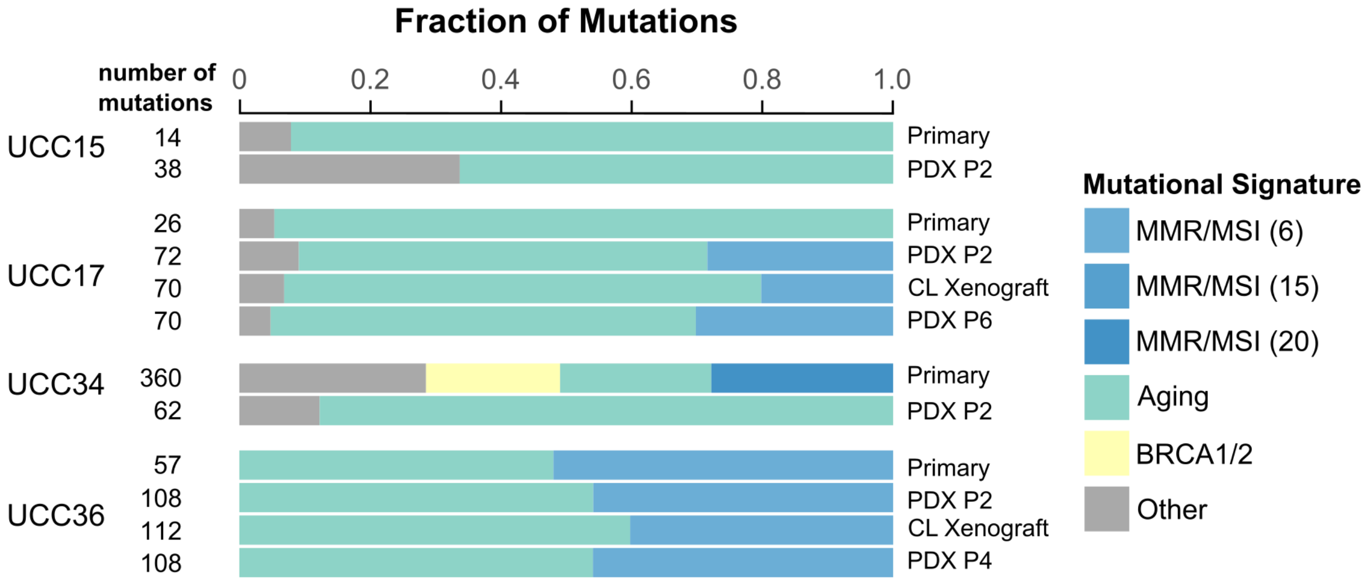

Supplementary Fig. 4 Mutation signature decomposition analysis for the four MSI-H patient tumor/xenograft model pairs. MSI signatures accounting for more than 20% of the mutations identified are shown.

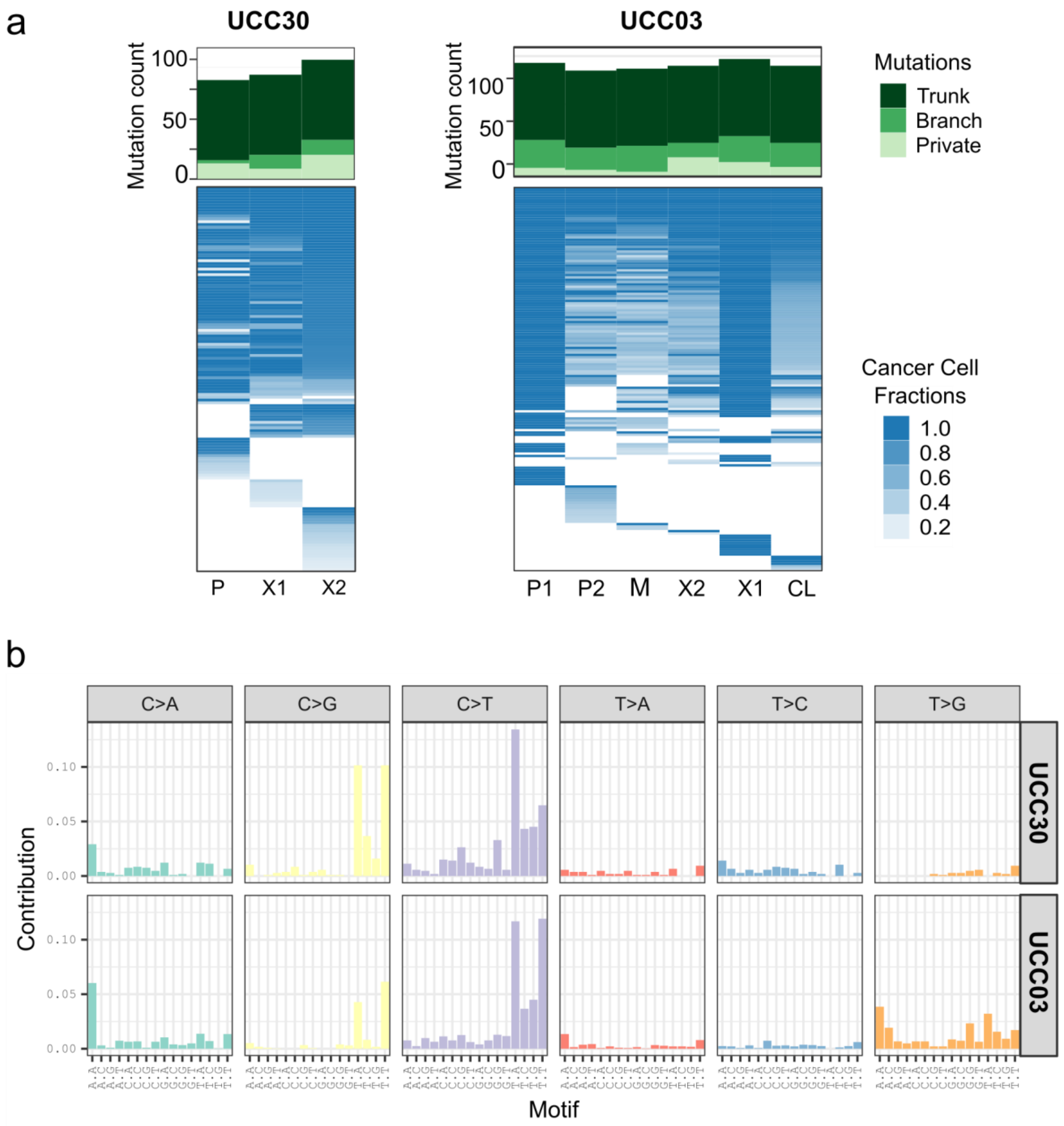

Supplementary Fig. 5 Mutational concordance by WES of the UCC30 and UCC03 models. **a** For the samples shown in Fig. 2d, mutations were divided into truncal mutations shared across all samples derived for the specified patient, private mutations unique to individual samples and branch mutations that were shared by some but not all samples from the specified patient. **b** Related to Fig. 2e. SNP mutation patterns of the combined results from each case.

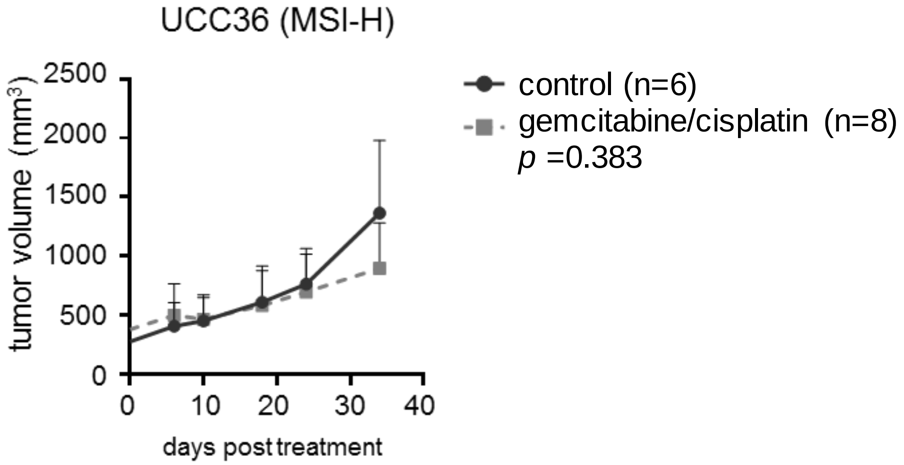

Supplementary Fig 6. Chemosensitivity of the MSI-H UCC36 PDX model. The PDX model was treated with either the combination of gemcitabine and cisplatin (gray square) or vehicle (black circle) only as control (p=0.383). Two-way ANOVA test (Prism) was used for statistical analysis without adjustment. Data are presented as mean values +/- SD.
