# Supplementary material for: Modeling biological and genetic diversity in upper tract urothelial carcinoma with patient derived xenografts
Source: Nat Commun. 2020 Apr 24;11:1975. doi: 10.1038/s41467-020-15885-7 (PMC7181640; doi:10.1038/s41467-020-15885-7)
Supplement: Supplementary file 2 — Reporting Summary [file 41467_2020_15885_MOESM2_ESM.pdf]

nature research

Corresponding author(s): David B Solit
Jonathan A Coleman

Last updated by author(s): Mar 5, 2020

Reporting Summary

Nature Research wishes to improve the reproducibility of the work that we publish. This form provides structure for consistency and transparency in reporting. For further information on Nature Research policies, see Authors & Referees and the Editorial Policy Checklist .

Statistics

For all statistical analyses, confirm that the following items are present in the figure legend, table legend, main text, or Methods section.

n/a	Confirmed	
☐	☒	The exact sample size (n) for each experimental group/condition, given as a discrete number and unit of measurement
☐	☒	A statement on whether measurements were taken from distinct samples or whether the same sample was measured repeatedly
☐	☒	The statistical test(s) used AND whether they are one- or two-sided<br>*Only common tests should be described solely by name; describe more complex techniques in the Methods section.*
☐	☒	A description of all covariates tested
☐	☒	A description of any assumptions or corrections, such as tests of normality and adjustment for multiple comparisons
☐	☒	A full description of the statistical parameters including central tendency (e.g. means) or other basic estimates (e.g. regression coefficient) AND variation (e.g. standard deviation) or associated estimates of uncertainty (e.g. confidence intervals)
☐	☒	For null hypothesis testing, the test statistic (e.g. F, t, r) with confidence intervals, effect sizes, degrees of freedom and P value noted<br>*Give P values as exact values whenever suitable.*
☒	☐	For Bayesian analysis, information on the choice of priors and Markov chain Monte Carlo settings
☐	☒	For hierarchical and complex designs, identification of the appropriate level for tests and full reporting of outcomes
☒	☐	Estimates of effect sizes (e.g. Cohen's d, Pearson's r), indicating how they were calculated

Our web collection on statistics for biologists contains articles on many of the points above.

Software and code

Policy information about availability of computer code

Data collection	No software was used for data collection
Data analysis	RNA-Seq and DNA mutation sequencing data were first analyzed using the pipelines in our institution for reads per gene and mutation callings. R/Bioconductor packages were then used to analyze RNA-Seq data (DESeq2 package) and DNA mutations (maftools package). In addition, gene set variation analysis was performed using single sample GSEA (within GSVA package). Extracellular matrix properties were analyzed by Zeiss Zen Imaging and FIJI (Image J) software. GraphPad Prism v8 software was used to analyze in vivo tumor growth studies. consensusMIBC package (cit-bioinfo, version 1.1.0) was used for a consensus clustering analysis.

For manuscripts utilizing custom algorithms or software that are central to the research but not yet described in published literature, software must be made available to editors/reviewers. We strongly encourage code deposition in a community repository (e.g. GitHub). See the Nature Research guidelines for submitting code & software for further information.

Data

Policy information about availability of data

All manuscripts must include a data availability statement. This statement should provide the following information, where applicable:

- Accession codes, unique identifiers, or web links for publicly available datasets
- A list of figures that have associated raw data
- A description of any restrictions on data availability

All genomic data are available on cBioPortal with the link (https://www.cbioportal.org/study/summary?id=utuc_msk_2019; https://www.cbioportal.org/study/summary?id=utuc pdx_msk_2019). The raw and normalized RNA sequencing data are available in the database of Gene Expression Omnibus (GEO) associated with series entry number GSE134292. The source data underlying Figs. 1a-b, 2b-d, 3b-d, 4a-c, 5c-e and Supplementary Figs. 2, 4, 6 are provided as a Source Data File.

Field-specific reporting

Please select the one below that is the best fit for your research. If you are not sure, read the appropriate sections before making your selection.

☒ Life sciences ☐ Behavioural & social sciences ☐ Ecological, evolutionary & environmental sciences

For a reference copy of the document with all sections, see nature.com/documents/nr-reporting-summary-flat.pdf

Life sciences study design

All studies must disclose on these points even when the disclosure is negative.

Sample size

34 tumor samples from 34 corresponding UTUC (MSKCC) patients at MSKCC were included in the study of generating patient-derived xenograft models. MSK-IMPACT analysis was performed for all 34 patient tumors, matching normal tissue (or blood) and all matching patient-derived models. RNAseq was performed in 80 UTUC patient tumors which passed the quality control of library preparation among 117 initial cohorts.

Per in vivo animal studies:
We performed a power analysis to predict the minimum number of animals required to obtain a statistically significant result, given our preliminary assumptions of the difference between groups and expected variances. We used the following equation for continuous variables:
n = [(1/q1=1/q2)S2(zalpha + zbeta)2]E2
where n is the required sample size, q1 is the proportion in group 1 (0.5), q2 is the proportion in group 2 (0.5), S is the estimated standard error of the mean for the two groups, zalpha is the standard normal deviate for the alpha (1.96 when alpha is 0.05 or 95% CI), zbeta is the standard normal deviate for beta (0.84 when beta is 0.8 or 80%), and E is the estimated difference between the means. Based on this analysis, we estimate the need for 8 animals per group in each experiment.

Base on this analysis, we have initiated studies with at least 8 mice per group. However, due to mortality during the experimental period, the number of mice for two experimental groups fall below 8.

Data exclusions

No data were excluded

Replication

In vitro cell growth assays and western blot analyses were repeated at least 3 times with reproducibility. Several methods were used for each analysis; for example for detection of UTUC subtypes, 2 different classifiers were used.

Randomization

Animal allocation bearing tumors per each experimental group was randomized based on tumor volume. Randomization is not applicable to the studies using patient samples.

Blinding

As one staff was treating and monitoring animals, in vivo experiments were not blinded. Blinding is not applicable to the studies using patient samples.

Reporting for specific materials, systems and methods

We require information from authors about some types of materials, experimental systems and methods used in many studies. Here, indicate whether each material, system or method listed is relevant to your study. If you are not sure if a list item applies to your research, read the appropriate section before selecting a response.

Materials & experimental systems

n/a	Involved in the study
☐	☒ Antibodies
☐	☒ Eukaryotic cell lines
☒	☐ Palaeontology
☐	☒ Animals and other organisms
☐	☒ Human research participants
☐	☒ Clinical data

Methods

n/a	Involved in the study
☒	☐ ChIP-seq
☒	☐ Flow cytometry
☒	☐ MRI-based neuroimaging

Antibodies

Antibodies used

ERK (#9102), p-ERK (T202/Y204) (#9101), AKT (#9272), p-AKT (Ser473) (#9271) antibodies were from Cell Signaling Technology.

Validation

ERK (#9102) citations:
1. Khan, F. U., Owusu-Tieku, N. Y. G., et al. (2019), 'Wnt/β-Catenin Pathway-Regulated Fibromodulin Expression Is Crucial for Breast Cancer Metastasis and Inhibited by Aspirin.', Front Pharmacol, 10, pp. 1308
2. Fujiwara, H., Tateishi, K., et al. (2019), 'Mutant IDH1 confers resistance to energy stress in normal biliary cells through PFKP-induced aerobic glycolysis and AMPK activation.', Sci Rep, 9 (1), pp. 18859

AKT (#9272) citations:
1. Liu, J., Liang, H., et al. (2019), 'Ivermectin induces autophagy-mediated cell death through the AKT/mTOR signaling pathway in glioma cells.', Biosci Rep, 39 (12)
2. Fujiwara, H., Tateishi, K., et al. (2019), 'Mutant IDH1 confers resistance to energy stress in normal biliary cells through PFKP-induced aerobic glycolysis and AMPK activation.', Sci Rep, 9 (1), pp. 18859

p-AKT (Ser473) (#9271) citations:
1. Ding, X., Ge, L., et al. (2019), 'Docosahexaenoic Acid Serving As Sensitizing Agents And Gefitinib Resistance Revertants In EGFR Targeting Treatment.', Onco Targets Ther, 12, pp. 10547-10558
2. Zhang, S., Xu, J., et al. (2019), 'Sodium Selenate Ameliorates Cardiac Injury Developed from High-Fat Diet in Mice through Regulation of Autophagy Activity'., Sci Rep, 9 (1), pp. 18752

p-ERK (T202/Y204) (#9101) citations:
1. Fujiwara, H., Tateishi, K., et al. (2019), 'Mutant IDH1 confers resistance to energy stress in normal biliary cells through PFKP-induced aerobic glycolysis and AMPK activation.', Sci Rep, 9 (1), pp. 18859
2. Wang, L., Tan, T. K., et al. (2019), 'ASCL1 is a MYCN- and LMO1-dependent member of the adrenergic neuroblastoma core regulatory circuitry.', Nat Commun, 10 (1), pp. 5622

Eukaryotic cell lines

Policy information about cell lines

Cell line source(s)	BT474 (ATCC), UCC14-PDC (a new line generated for the current work by the authors), CVX-4 (Dr Alessandro Santin, USA), MGH-U3 (Dr Margaret Knowles, UK)
Authentication	BT474 were authenticated by STR profiling with 100% match. UCC14-PDC, CVX-4 and MGH-U3 have unique STR profile.
Mycoplasma contamination	all cell lines tested negative for mycoplasma
Commonly misidentified lines (See ICLAC register)	None

Animals and other organisms

Policy information about studies involving animals; ARRIVE guidelines recommended for reporting animal research

Laboratory animals	mouse, NOD-SCID IL2Rg−/− (NSG) mice, male, 6-8 weeks old
Wild animals	None
Field-collected samples	None
Ethics oversight	the MSKCC Institutional Animal Care and Use Committee

Note that full information on the approval of the study protocol must also be provided in the manuscript.

Human research participants

Policy information about studies involving human research participants

Population characteristics	Patients from a single institution (Memorial Sloan Kettering Cancer Center) were recruited for participation in a prospective institution-wide tumor biobanking and sequencing effort. As a tertiary referral center specializing in cancer care we do see a higher volume of rare cancers such as upper tract urothelial cancer. Clinical data for the cohort of patients included in this study are seen in Table 1.
Recruitment	The protocol was offered to all patients presenting to the institution with upper tract urothelial carcinoma. As a tertiary referral center specializing in cancer care we do see a higher volume of UTUC than other institutions. Our patient population reflects the surrounding area of the institution but there is always possible selection bias with regards to ethnicity and socioeconomic status as a tertiary referral center. Regardless this study is on modeling with patient-derived xenografts and the higher volume of UTUC cases at our institution made this project feasible.
Ethics oversight	Memorial Sloan Kettering Cancer Center

Note that full information on the approval of the study protocol must also be provided in the manuscript.

Clinical data

Policy information about clinical studies

All manuscripts should comply with the ICMJE guidelines for publication of clinical research and a completed CONSORT checklist must be included with all submissions.

Clinical trial registration	NCT01775072 (MSK IRB #12-245), MSKCC IRB #89-076/06-107
Study protocol	NCT01775072 (MSK IRB #12-245) for molecular profiling of tumors, MSKCC IRB #89-076/06-107 for collection and research use of human tissue
Data collection	The study was approved by our Institutional Review Board (Memorial Sloan Kettering Cancer Center) IRB protocols for tumor biobanking – IRB #12-245. Banked excess tissue was collected from nephroureterectomy specimens, metastatic biopsies and endoscopic UTUC biopsies of patients with high-grade UTUC. UTUC samples were obtained from patients under protocols from March 2014 to April 2017 from Memorial Sloan Kettering Cancer Center. All pathology specimens were reviewed and reported by board-certified genitourinary pathologists in the Department of Pathology at MSKCC. Clinical charts were reviewed by the authors (N.A., T.N.C.) to record patient demographics, tobacco use, treatment history, anatomic site, presence of prior or concurrent bladder cancer, pathologic grade and stage using tumor, node, metastasis (TNM) system. RECIST evaluation of radiologic images were reviewed by a board-certified genitourinary radiology (H.A.V.)
Outcomes	While this is not a randomized clinical trial our outcome of interest was to establish patient derived xenografts and cell line models that reflect the genomic and biological heterogeneity of urothelial carcinoma.
